# Quality of Late-Life Depression Information on the Internet: Website Evaluation Study

**DOI:** 10.2196/36177

**Published:** 2022-09-12

**Authors:** Teaghan A M Pryor, Kristin A Reynolds, Paige L Kirby, Matthew T Bernstein

**Affiliations:** 1 Department of Psychology University of Manitoba Winnipeg, MB Canada; 2 Department of Psychiatry University of Manitoba Winnipeg, MB Canada

**Keywords:** late-life, depression, older adults, internet, websites, information quality, usability, readability

## Abstract

**Background:**

The internet can increase the accessibility of mental health information and improve the mental health literacy of older adults. The quality of mental health information on the internet can be inaccurate or biased, leading to misinformation.

**Objective:**

This study aims to evaluate the quality, usability, and readability of websites providing information concerning depression in later life.

**Methods:**

Websites were identified through a Google search and evaluated by assessing quality (DISCERN), usability (Patient Education Materials Assessment Tool), and readability (Simple Measure of Gobbledygook).

**Results:**

The overall quality of late-life depression websites (N=19) was adequate, and the usability and readability were poor. No significant relationship was found between the quality and readability of the websites.

**Conclusions:**

The websites can be improved by enhancing information quality, usability, and readability related to late-life depression. The use of high-quality websites may improve mental health literacy and shared treatment decision-making for older adults.

## Introduction

### Background

Late-life depression (LLD) can occur in adults aged ≥65 years, either for the first time or as a recurrent episode [1]. Major depressive disorder (MDD) is characterized by low mood or loss of interest in daily activities, changes in weight or appetite, trouble sleeping or sleeping too much, lack of energy, feelings of worthlessness or guilt, psychomotor agitation or retardation, difficulty focusing or making decisions, and thoughts of death or suicide. At least five of these symptoms must occur for most of the day, nearly every day, or for a period of at least 2 weeks [2].

Approximately 2%-6% of adults aged ≥55 years have received a diagnosis of MDD or experienced a major depressive episode within the past year [3-5]. The prevalence of MDD in older adults may be higher when including individuals experiencing subclinical depression [6,7]. Furthermore, the severity of depressive symptoms among older adults is particularly concerning, given that adults aged ≥70 years, and older men, in particular, have the highest rates of completed suicide worldwide [8].

It is important to note that older adults experiencing LLD can differ in symptomology compared with younger adults, such as presenting with fewer affective symptoms (eg, tearfulness), increased complaints of somatic symptoms, cognitive changes, and loss of interest [9]. Older adults are also more likely to experience comorbid health conditions and neurological disorders, which further affects the identification of LLD and influences the need for specialized treatment approaches [1,9].

When older adults experience a mental health problem, such as depression, they are faced with a lack of information regarding the symptoms and how to manage them with effective treatment options [10,11]. This gap in knowledge leads to lower levels of mental health literacy (knowledge about recognition, prevention, and management), which can complicate or delay the mental health treatment–seeking process [12,13]. Despite such barriers, most older adults have positive feelings about seeking help for mental health problems and a desire for increased information and participation in the treatment decision-making process [14,15].

Shared decision-making is a process that occurs between a patient and a health care provider, where diverse treatment options are shared by both parties to foster the agreement and implementation of a preferred treatment option [16]. Engaging in this process is beneficial for individuals with mental health problems, increasing their satisfaction and involvement in treatment decisions [17,18].

The internet can be a valuable tool for meeting the information needs of older adults. By presenting a wide variety of treatment options, web-based information can facilitate engagement in the shared treatment decision-making process [19]. As noted previously, web-based depression information should reflect the differences in symptom presentation and treatment options that are relevant to the unique needs of the older adult population [1].

Most internet users (58%-78%) use the internet to search for health information [20-25], and it is increasingly being used to access mental health information [26-29]. Older adults have high rates of internet use, with 73% of older adults using the internet [30,31] and 40% using the internet to access health information [32]. Little is known about specific internet use by older adults for mental health information, although a study found that 11% of older adults used it for finding information on mental health problems and expressed interest in using the internet as a tool for managing their mental health [33]. A more recent study found that 67% of adults with bipolar disorder aged ≥60 years who used the internet used it to access information about their disorder [34]. Despite this, some older adults feel as though they lack the knowledge and confidence to use the internet as a source of information [35].

Therefore, caregivers of older adults are often involved in seeking information for their care recipient and play an invaluable role in facilitating the treatment-seeking and shared decision-making process [36,37]. Part of the information-seeking process involves using the internet to access essential treatment information. Recent research has shown that a high percentage of caregivers (67%-71%) use the internet to access health information on behalf of the individual they support [38,39], particularly for older adults [40].

Despite the benefits of internet use for health information queries, including anonymity and accessibility, there are some disadvantages [41]. One of the drawbacks is the uncertainty of the quality of the information provided on the internet [31,42]. It may be difficult for individuals to determine whether the information presented is unbiased, accurate, and evidence based [43-46]. Inaccurate health information retrieved from the internet, which patients incorporate into their clinical requests, has been demonstrated to harm the physician-patient relationship and have detrimental effects on their health outcomes [47]. Therefore, evaluating the quality of the information provided on the web is essential in preventing the spread of misinformation and facilitating increased knowledge of balanced treatment options not only for older adults themselves but also for those who seek out information on their behalf.

As a result, studies evaluating website quality are increasing [48] as internet use for mental health information becomes more prevalent. Furthermore, studies have raised concerns regarding the approach that researchers have taken to evaluate websites and the lack of consistency across studies. An important question stemming from this growing research base is what constitutes a high-quality website. Common criteria used in the literature to identify high-quality websites are based on the principles of quality, usability, and readability [48-50]. Website quality involves the extent to which a website provides clear, unbiased, evidence-based information regarding mental health diagnoses and treatment options [51,52]. Usability characteristics refer to the ease of use, navigation, and aesthetics of websites [53], and readability is the ease of reading written text [54-56], both of which contribute to the overall quality of web-based information. Therefore, high-quality websites should be relatively straightforward to navigate and comprehend.

Website evaluations have been completed on a variety of health topics, including depression in the adult population [57-61]. A systematic review of studies evaluating the quality of mental health websites found that 23 of the 31 studies had poor quality overall [48]. A small number of studies have been conducted assessing the quality of depression information provided by websites. Generally, studies have shown that the quality of website information is poor [57-59]. However, one study examining the overall quality of websites for depression in adults aged 18 to 64 years found adequate quality, with most websites scoring higher than the mean score on a measure of content quality [61]. The discrepancy in findings could be because of variability in the website evaluation methodology used across studies. Nevertheless, this range of quality (poor to adequate) may not be helpful to consumers.

### Objectives

Upon extensive review of the website evaluation literature, to the best of our knowledge, no study has examined the quality of websites specific to depression that appears or worsens in later life (LLD). Given the unique impact of aging on depression presentation, the involvement of caregivers in the treatment-seeking processes, and the influence of web-based information in the shared decision-making process, we deemed it essential to evaluate the quality of websites providing information on LLD. Websites were evaluated according to (1) quality of information, as evaluated by DISCERN [62], a standardized measure of website quality; (2) usability, as determined by the Patient Education Materials Assessment Tool (PEMAT) [63]; and (3) readability of information, as evaluated by the Simple Measure of Gobbledygook (SMOG) [64]. A secondary objective of this study was to determine whether website quality was related to usability and readability.

On the basis of the existing mental health website evaluation literature, we hypothesized that (1) website quality would be adequate to poor according to DISCERN evaluations, (2) usability would be adequate to poor, and (3) the reading level would be higher than the recommended levels for health information (grades 7-8). Furthermore, we hypothesized that website quality, usability, and readability would be related. Specifically, the quality of websites would be positively associated with usability scores and negatively associated with reading levels.

## Methods

### Website Selection

Google Canada search engine was used to identify websites as it is the most widely used search engine worldwide [65] and has been identified as the starting point for most internet users seeking health information [30,42,66]. The search was completed on one of the computers in the research laboratory by the primary investigator (TAMP), where website cookies and search history were cleared before searching to prevent influencing the search results. The search terms “older” AND “depression” were used to complete the initial search to target websites presenting distinct information on LLD in this age group (presentation and treatment options).

Websites within the first 3 pages of the search were evaluated if they did not meet exclusion criteria, as it has been found that most search engine users do not go past the first 3 pages of the search [67]. Websites were excluded from the evaluation if they were advertising or selling products; presented information from books or articles, as the purpose of this study is to evaluate websites; contained minimal information (<500 words) that was not substantial enough to evaluate; provided information that was not focused on LLD; and were not written in English, as this is the researchers’ language of origin. The website selection method and exclusion criteria were in line with prior depression website evaluation research [57-60].

### Procedure

#### Measures of Website Quality

The DISCERN instrument is a standardized measure comprising 16 items assessing the quality of written health information [62] and has been used in a variety of health website evaluation studies [68-71]. The reliability of DISCERN has been psychometrically evaluated in previous research [72-74] and is able to differentiate between low- and high-quality information [74]. DISCERN comprises 3 main sections focusing on how reliable the publication is, the quality of information for treatment options, and the overall quality of the publication [62]. Each item is rated on a 5-point scale from no to yes indicating the extent to which the information fulfills the criteria: 1 (*criterion was not fulfilled at all*), 2 to 4 (*criterion was fulfilled to some extent*), and 5 (*criterion was completely fulfilled*).

#### Usability

The PEMAT was used to assess usability. The PEMAT is a multi-item standardized tool used to assess the understandability (ability to understand materials) and actionability (ability to encourage consumers to take action on information presented) of materials educating patients on a variety of health topics [63]. The PEMAT has been used in numerous recent studies evaluating printed and web-based health-related materials [75,76]. The tool has been evaluated and found to have good internal consistency, reliability, and construct validity [77,78]. The tool is divided into 2 domains—understandability and actionability—with specific topic areas under each domain. The number of items used varies depending on the type of material used, either printed or audiovisual. For this study, PEMAT-printed was used, which comprises 34 items (17 understandability and 7 actionability items), as most of the website information can be printed from each website. Information was rated according to each item and scored either 0=*disagree* or 1=*agree*, with some items having the option of *not applicable*=NA. Separate usability and actionability scores were calculated by summing the total number of points given (excluding *not applicable* items) and dividing by the total number of possible points. This number was multiplied by 100 to obtain a percentage score to determine what percentage of the material is understandable or actionable.

#### Readability

A readability score was calculated for each website using the SMOG. The SMOG assesses the number of words with ≥3 syllables in 10 consecutive sentences sampled from the beginning, middle, and end of the text [79]. Although the Flesh-Kinkaid reading formula has been most frequently used and cited in assessing the readability of health information, the SMOG formula is recommended as a more appropriate formula to assess health information because of its consistency and ease of use when calculating reading level [79].

### Analyses

The analysis component comprised (1) descriptive, (2) correlational, and (3) inferential statistics. All statistics were computed by the first author (TAMP) using SPSS (version 21.0; IBM Corp) for Windows. Descriptive analyses produced a mean score for each website, as well as a mean score and 95% CI for each DISCERN item. In addition, 2 percentages were computed for the domains of understandability and actionability according to the PEMAT. A 2-tailed Pearson correlation was calculated to determine the relationship between website quality (as determined by DISCERN) and website usability scores (as determined by the PEMAT) and between website quality and website reading level independently. An intraclass correlation coefficient was computed between the first half of the selected websites to determine the level of agreement between the primary (TAMP) and secondary (PLK) raters for DISCERN and the PEMAT, similar to previous research [60,80].

### Ethical Considerations

This research did not involve human participants and involved the examination of mental health information in the public domain. Therefore, it was determined that no ethics approval was required to carry out this study.

## Results

### Website Characteristics

Evaluated websites (N=19) are described in Table 1. This sample is consistent with previous research [57-60]. Most of the websites were from the United States; however, websites from Australia (Beyond Blue), Canada (HealthLinkBC), and Great Britain (Royal College of Psychiatry) also emerged in the initial search. The evaluated websites were hosted by hospitals (eg, Johns Hopkins Medicine), nonprofit organizations (Age UK and HelpGuide), and government organizations (National Institute of Mental Health). Most of the websites did not have a formal operationalized definition of LLD and used a variety of terms when defining LLD (eg, elderly or geriatric depression, depression and older adults or in older people, and aging and depression). Websites identified the ages of 60 to ≥65 years as a target age group within the late-life period.

**Table 1:** Late-life depression website characteristics.

Website (country)	Search engine order	Overall quality^a,b^	Usability^c^^,d^		Readability^e^
			Understandability score (%)	Actionability score (%)	
Age UK (United Kingdom)	24	2.9	69.2	60	9.5
American Psychological Association (United States)	12	2.3	53.8	40	11.1
Beyond Blue (Australia)	4	4.1	53.8	60	12.5
Black Dog Institute (Australia)	16	2.5	76.9	20	12.4
Canadian Coalition for Senior’s Mental Health (Canada)	28	3.0	84.6	60	6.9
Centers for Disease Control and Prevention (United States)	10	2.3	46.2	40	10.7
Health in Aging (United States)	17	4.0	53.8	60	11.5
Healthline (United States)	11	3.6	76.9	60	10.8
HealthLinkBC (Canada)	30	3.9	76.9	80	7.4
HelpGuide (United States)	1	3.4	71.4	60	9.6
Johns Hopkins Medicine (United States)	13	2.6	69.2	40	10.9
MedlinePlus (United States)	9	2.3	66.7	40	8.8
Mental Health America (United States)	5	3.4	61.5	80	11.2
National Institute of Mental Health (United States)	3	3.9	75.0	40	10.2
National Institute on Aging (United States)	2	3.3	53.8	60	10.6
Royal College of Psychiatrists (United Kingdom)	8	4.8	69.2	50	7.1
Substance Abuse and Mental Health Services (United States)	29	3.9	57.1	20	12.4
WebMD (United States)	6	3.6	46.2	40	9.8
World Health Organization	25	1.5	30.8	0	13.5

^a^Overall quality was measured by DISCERN.

^b^The mean DISCERN score is a 1 to 5 rating averaged across 16 items.

^c^Usability was measured by the Patient Education Materials Assessment Tool.

^d^To calculate the scores, items that are agreed upon are summed and divided by the total possible points then multiplied by 100 to get a percentage.

^e^Readability was measured by the Simple Measure of Gobbledygook.

### Website Quality

A mean score was provided for each website to better understand the quality of the information presented. Website quality varied greatly, with scores ranging from 1.5 (low quality) to 4.8 (high quality) out of a total score of 5 (Table 1). Websites that scored highly on DISCERN included *the Royal College of Psychiatrists* (4.8), *Beyond Blue* (4.1), and *Health in Aging* (4.0).

An average score across the websites was also computed for each DISCERN item to better understand the criteria that were well addressed and criteria that needed improvement (Table 2). Most of the websites addressed the DISCERN items moderately well, with average DISCERN scores ranging from 2.4 to 4.0 (SD 1.0 to 1.8). The mean score of item 16, which served as an overall rating of the websites, was 3.2. This indicates that websites with information about LLD were of adequate quality. According to scores on specific DISCERN criteria, websites clearly showed that there were multiple treatment options available (average score of 4.0). Information presented by websites was relevant to the older adult population (average score of 3.7). Websites also encouraged shared decision-making with health care providers or family members (average score of 3.7). Websites lacked information on the risks of treatment options (average score of 2.4), describing how each treatment worked (average score of 2.5), and the benefits of each treatment (average score of 2.6). Most websites did not provide the sources used to create the publication (average score of 2.6). An intraclass correlation was computed to determine the reliability of the raters on the DISCERN measure, which determined that there was an excellent level of agreement (*r*_9_=0.90; *P*<.001).

**Table 2:** Mean scores of DISCERN items across all websites^a^.

Item number	DISCERN item	Score, mean (SD; 95% CI)
1	Are the aims clear?	3.4 (1.1; 2.9-3.9)
2	Does it achieve its aims?	3.6 (1.0; 3.2-4.1)
3	Is it relevant?	3.7 (1.2; 3.2-4.2)
4	Is it clear what sources of information were used to compile the publication (other than the author or producer)?	2.6 (1.6; 1.9-3.3)
5	Is it clear when the information used or reported in the publication was produced?	3.3 (1.3; 2.7-3.9)
6	Is it balanced and unbiased?	3.6 (1.0; 3.1-4.0)
7	Does it provide details of additional sources of support and information?	3.2 (1.4; 2.6-3.9)
8	Does it refer to areas of uncertainty?	3.1 (1.3; 2.5-3.7)
9	Does it describe how each treatment works?	2.5 (1.2; 2.0-3.1)
10	Does it describe the benefits of each treatment?	2.6 (1.2; 2.1-3.2)
11	Does it describe the risks of each treatment?	2.4 (1.3; 1.8-3.0)
12	Does it describe what would happen if no treatment was used?	2.8 (1.8; 2.0-3.6)
13	Does it describe how the treatment choices affect the overall quality of life?	3.4 (1.0; 3.0-3.9)
14	Is it clear that there may be more than one possible treatment choice?	4 (1.3; 3.4-4.6)
15	Does it provide support for shared decision-making?	3.7 (1.2; 3.2-4.3)
16	Based on the answers to all of the above questions, rate the overall quality of the publication as a source of information about treatment choices.	3.2 (1.2; 2.6-3.7)

^a^Each DISCERN item is rated on a 5-point scale with the anchors 1=did not meet criteria and 5=did meet criteria.

### Usability

The understandability scores of the PEMAT (Table 1) varied, ranging from 30.8% to 84.6% (mean 62.8%). Only 32% (6/19) of websites met the 70% criteria, indicating that the website was understandable [77]. With regard to specific understandability criteria, most websites used an active voice and used a variety of visual cues to draw attention to important points of the websites. Websites lacked summaries of information and introduced complex medical terms within the text without definition. In examining the actionability section of the PEMAT, websites presented a range of scores from 0% to 80% (mean 47.9%), with only 10% (2/19) of the 19 websites meeting the minimum 70% threshold for websites to be deemed actionable. Upon further examination, most websites identified at least one action that individuals could take (eg, talking to their physician). Despite this, most websites did not provide any visual aids encouraging individuals to take action, lacked tools to aid individuals in taking action (eg, treatment planning sheet), and did not break down suggested actions into explicit steps.

Two separate correlations were computed between the mean DISCERN scores and the usability and actionability percentages of the PEMAT to determine the relationship between website quality and usability. The correlation between DISCERN scores and the understandability scores of the PEMAT was not significant (*r*_17_=−0.30; *P*=.21). By contrast, the correlation between the DISCERN scores and the actionability scores of the PEMAT was found to be significant (*r*_17_=0.49; *P*=.04). An intraclass correlation was also computed to determine the interrater reliability, which established that there was an excellent level of agreement for the understandability section (*r*_9_=0.90; *P*<.001).

### Readability

The readability of the websites was calculated using the SMOG readability formula, which produced a grade level score. The reading levels of the websites ranged from 6.9 to 13.5, with an average grade level of 10.4 across all websites (Table 1). Only 16% (3/19) of the websites met the National Institute of Health’s recommended grade level (grade 7-8). A correlational analysis was conducted to determine whether website readability was related to website quality, as measured by DISCERN. This analysis was nonsignificant (*r*_17_=−0.31; *P*=.20).

### Website Dimension Comparison

Table 3 provides a simplified dimension description (good, adequate, or poor) for each website based on the evaluation measure scores, defined differently for each dimension: quality where good≥4, adequate=3 to 4, and poor≤3 (mean 1-5 rating scale); usability where good≥80, adequate=70 to 80, and poor≤70 (percentage understandable or actionable); readability, where good≤10, adequate=10 to 12, and poor≥12 (grade levels; Table 3). The rationale behind the cutoffs for these quality dimensions was based on how difficult it was for the websites to attain the recommended levels for each measure. For website quality, *Good* was used to describe websites that received a rating of >4 on the DISCERN measure as most websites were not able to achieve this. Similarly, with readability, most websites were not able to meet the recommended reading level (grade 7-8); thus, they were rated *Good* if they achieved a reading level of <10.

**Table 3:** Website dimension comparison^a^.

Website (country)	Search engine order	Overall quality^b^	Usability^c^		Readability^d^
			Understandability score	Actionability score	
HelpGuide (United States)	1	Adequate	Adequate	Poor	Good
National Institute on Aging (United States)	2	Adequate	Poor	Poor	Adequate
National Institute of Mental Health (United States)	3	Adequate	Adequate	Poor	Adequate
Beyond Blue (Australia)	4	Good	Poor	Poor	Poor
Mental Health America (United States)	5	Adequate	Poor	Good	Adequate
WebMD (United States)	6	Adequate	Poor	Poor	Good
Royal College of Psychiatrists (United Kingdom)	8	Good	Poor	Poor	Good
MedlinePlus (United States)	9	Poor	Poor	Poor	Good
Centers for Disease Control and Prevention (United States)	10	Poor	Poor	Poor	Adequate
Healthline (United States)	11	Adequate	Adequate	Poor	Adequate
American Psychological Association (United States)	12	Poor	Poor	Poor	Adequate
Johns Hopkins Medicine (United States)	13	Poor	Poor	Poor	Adequate
Black Dog Institute (Australia)	16	Poor	Adequate	Poor	Poor
Health in Aging (United States)	17	Good	Poor	Poor	Adequate
Age UK (United Kingdom)	24	Poor	Poor	Poor	Good
World Health Organization	25	Poor	Poor	Poor	Poor
Canadian Coalition for Senior’s Mental Health (Canada)	28	Adequate	Good	Poor	Good
Substance Abuse and Mental Health Services (United States)	29	Adequate	Poor	Poor	Poor
HealthLinkBC (Canada)	30	Adequate	Adequate	Good	Good

^a^Each website was rated on each dimension as good, adequate, or poor, defined differently for each dimension.

^b^Overall quality was measured by the DISCERN, where good ≥4, adequate=3 to 4, and poor ≤3 (mean 1-5 rating scale).

^c^Usability was measured by the Patient Education Materials Assessment Tool, where good ≥80, adequate=70 to 80, and poor ≤70 (percentage understandable or actionable).

^d^Readability was measured by the Simple Measure of Gobbledygook, where good≥10, adequate=10 to 12, and poor ≤12 (grade levels).

## Discussion

### Principal Findings and Comparison With Prior Work

The purpose of this study was to evaluate the overall quality of LLD information provided by websites, as evaluated by standardized measures of website quality (DISCERN), usability (PEMAT), and readability (SMOG). A secondary purpose of this study was to examine the relationships among website quality, usability, and readability. Website quality ranged from low to high when examining the DISCERN mean scores. Furthermore, when looking at the average of the overall DISCERN rating (item 16), websites were of moderate quality (3.2/5), which indicates that LLD website quality is poor to adequate, providing support for the hypothesis that website quality would be poor to adequate. This finding is consistent with previous website evaluation studies [48,57-59,61].

There were several high-quality websites, with *the Royal College of Psychiatrists* being identified as the website presenting the highest quality information according to DISCERN (4.8). This website also obtained a recommended reading level (grade 7.1), although usability (69.2%) and actionability (50%) were poor. The *Royal College of Psychiatrists* clearly identified the aims of the website and the sources used to create the information according to DISCERN. The same cannot be said for general trends across websites as the mean DISCERN score indicating identification of sources (item 4; “Is it clear what sources of information were used to compile the publication?”) was one of the lowest scores. Sources or references used to create web-based information should be clearly identified as consumers perceive them as positive content indicators, highlighting the trustworthiness and transparency of web-based health information [81].

The *Royal College of Psychiatrists* provided relevant information on comorbid problems that occur late in life and how they might interact with depression, such as physical symptoms, long-term health problems, cognitive issues, and loneliness. This trend was also seen across other evaluated websites more generally, as they provided content that was highly relevant to depression in older adults (eg, sections addressing dementia, vascular depression, and insomnia), which was supported by one of the highest mean DISCERN scores evaluating relevance (item 3; “Is it relevant?”). Providing age-relevant depression information is particularly important as it can aid in increased knowledge about symptom recognition and differentiation of presenting problems (depression vs dementia), which has been shown to lead to more timely access to mental health services [82,83]. This finding is especially relevant for older adults in light of research demonstrating their lower levels of mental health literacy [84,85].

Furthermore, the *Royal College of Psychiatrists* provided a range of unbiased treatment options, such as talk, medication, and complementary and brain stimulation treatments. This trend was also observed across all websites according to the high mean DISCERN score, indicating that the content was unbiased (item 6; “Is it balanced and unbiased?”). The *Royal College of Psychiatrists* included specific information to encourage the involvement of caregivers, specifically how to identify depression, encourage help seeking, and improve communication between caregivers and recipients. More broadly, information to encourage shared decision-making (eg, sections about talking with physicians or how to help someone with depression) had one of the highest scores across websites according to the mean DISCERN score evaluating support for shared decision-making (item 15; “Does it provide support for shared decision-making?”).

This finding is important to note, as prior research supports older adults’ preference for involvement in decisions related to their health and treatment options [86], and caregivers have been identified as an important part of this process [36]. The inclusion of this information on websites could serve to encourage and improve the shared decision-making processes among older adults, caregivers, and health care providers [87]. As shown previously, websites that provide evidence-based information, diverse treatment options, and content that supports shared decision-making constitute a high-quality website [36,37,52].

Websites generally failed to provide high-quality information about how different treatments work and the risks and benefits associated with those treatment options. A key premise of the shared decision-making process is to provide patients with different treatment options and allow them to weigh the risks and benefits associated with those treatments [16]. Incomplete treatment information may bias the shared decision-making process and limit individuals’ ability to fully weigh their treatment options [88]. Treatment information that clearly defines how treatments work and the associated risks and benefits serve to enhance mental health literacy, consumer empowerment, and shared decision-making [88-90]. Furthermore, when consumers have timely access to balanced, evidence-based information, they can make treatment decisions that align with their preferences [91].

Most websites did not meet the minimum recommended levels of understandability and actionability. Websites with higher usability used formatting that facilitated better understanding, such as bolded main headings and subheadings, large fonts or the option to increase the font, information in short paragraphs or bullet points, boxes to highlight important information, and presenting most information on a single page. Despite providing formatting that promoted website usability, some websites had definitions of medical terms as hyperlinks, forcing individuals to navigate away from the original page the search brought them to. It was also difficult to locate treatment sections on certain websites (eg, *Beyond Blue* and *Mental Health America*), as they were not clearly marked or listed under the adult depression sections. These findings are an especially meaningful aspect of website quality for older adult populations as they may have different needs when using websites, such as the need for increased font size, darker letters with lighter backgrounds, short sections of information, and limited navigational steps when searching [92].

Websites identified actions that individuals could perform to engage in their depression treatment (eg, behavioral strategies); however, most did not go on to further explain the steps and how to complete the recommended actions, limiting consumers’ ability to take the next steps in the help-seeking process. Although the understandability section of the PEMAT was not significantly correlated with DISCERN, the actionability section was significantly positively correlated with DISCERN. Specifically, as the quality of information increased, the actionability of the provided information increased. This finding suggests that higher-quality websites included information that encourages individuals to take action regarding their treatment, ideally resulting in prompt access to mental health services.

Most websites did not meet the recommended reading level (grade 7-8), and therefore, the readability of the websites was poor overall, providing support for the hypothesis that the reading level will be higher than the recommended levels for health information (grades 7-8). This finding is in line with other web-based mental health information evaluation studies that have also found low readability levels among websites [36,48,50,54,60,93]. Website quality was not found to be associated with website readability. A possible explanation for this is that the websites used more complex medical terms to provide higher-quality information but at the cost of an increased reading level. This relationship has been observed in a recent study evaluating the reliability, readability, and quality of hip impingement information on the internet [94]. Furthermore, it is of interest that most of the websites failed to define complex medical terms. It is important to use simple and clear written content and provide definitions for more complex terms to make the content accessible to individuals at all reading levels.

### Limitations

Although this study addresses significant gaps in website evaluation research, there are a number of limitations to note. First, search terms may not be representative of all web-based search strategies used to access information on LLD. Second, the websites included in this study were found by a routine Google search and do not represent the entirety of the websites that could be available to provide information on LLD. Third, raters’ pre-existing knowledge about the quality of particular organizations’ websites may have also biased raters’ perceptions of the quality and usability of the measures used in this study.

### Practice Implications and Recommendations

This study identifies high-quality websites and provides valuable insights into which websites older adults and their caregivers should access to receive high-quality information about treatment options. It also highlights websites providers can recommend to their clients as a resource, such as the *Royal College of Psychiatrists*, *Beyond Blue*, and *Health in Aging*.

Consumers of LLD information should seek web-based resources that discuss the impact of comorbid health problems and associated treatment options specific to this population. Consumers are also encouraged to engage with websites that provide clear evidence-based sources, as the identified sources can be an indicator of transparency and trustworthiness. Older adults and caregivers should seek websites that include content encouraging engagement in shared decision-making, such as how to discuss treatment options with their physician or involve a caregiver in their treatment.

Finally, these findings provide guidance for organizations and website developers to consider when designing a website for older adults. Developers should consider using bolded headings, larger fonts, bullet points, short paragraphs, and text boxes to increase understandability and comprehension of the content. Moreover, developers should consider making navigation between adult depression pages and pages specific to older adults more cohesive, as well as sections of importance (eg, treatment sections) easy to find. Regarding content, developers should further break down actions into small measurable steps encouraging consumers to engage in behavioral changes or treatment seeking and provide tools to aid individuals in taking action (eg, treatment planning sheet). They should use simple and understandable language and provide in-text definitions or glossaries for more complex terms, subsequently increasing information accessibility to individuals with diverse educational backgrounds.

### Future Research

As more people use the internet to access information about mental health problems, it is imperative to understand the quality of websites on the internet to understand whether older adults, caregivers, and health care providers are accessing easy-to-use, accurate, and comprehensive resources. Future research should examine older adults’ search strategies and, more specifically, whether older adults identify themselves as “older” when searching for mental health information on the web. Regardless of how older adults identify themselves, websites should better structure how their information is categorized on their websites to ensure that older adults are accessing the information relevant to them.

It will also be important to look more in depth at the usability characteristics of websites that the PEMAT did not address. A measure such as the Visual Aesthetics of Websites Inventory, which examines more specific aesthetics of websites such as simplicity, diversity, colorfulness, and craftsmanship, would be useful in further evaluating usability [95]. Future researchers should fully evaluate the aesthetics of websites as it has implications on individuals’ first impressions and whether they will revisit the site or recommend it to others [96].

### Conclusions

This is the first study to examine the quality of LLD information on the internet, and it addresses a gap in the literature by highlighting the quality of several LLD websites accessible on the internet. This study took a multifaceted approach to measuring website quality by using multiple measures to better understand different aspects that contribute to the overall quality of websites. The quality of LLD websites varied, ranging from low to high quality. Overall, the quality of the websites was adequate, and the usability and reading levels of the websites were poor. Websites provided information about particular problems that may affect depression in later life but lacked key information on how treatments work and the risks and benefits associated with treatments. Treatment sections were difficult to navigate or were found under adult depression sections. The ability of the websites to encourage understanding and action in individuals was also poor, and the information presented was higher than the recommended reading level. Websites were strong at providing multiple treatment options relevant to older adults and encouraging shared decision-making. They provided visual cues and formatting, which facilitated better understanding (eg, use of bolded headings, short paragraphs, or bullet points), and some websites were able to attain the recommended reading level. Website developers should consider increasing the quality, usability, and readability to produce high-quality information for older adults. High-quality websites may increase the mental health literacy of older adults and caregivers and improve the shared decision-making process. Health care providers should be aware of high-quality websites and should incorporate the use of high-quality websites into the shared decision-making process. They should direct older adults and caregivers to the high-quality websites identified in this study and use them as a decision-making tool by directing them to sections presenting different treatment options to further discuss in their ongoing care.

## Abbreviations

LLD: late-life depression
MDD: major depressive disorder
PEMAT: Patient Education Materials Assessment Tool
SMOG: Simple Measure of Gobbledygook
